# Neural and behavioral evidence supporting the relationship between habitual exercise and working memory precision in healthy young adults

**DOI:** 10.3389/fnins.2023.1146465

**Published:** 2023-04-06

**Authors:** Xuye Yuan, Dongwei Li, Yiqing Hu, Mengdi Qi, Yuanjun Kong, Chenguang Zhao, Jing Huang, Yan Song

**Affiliations:** ^1^State Key Laboratory of Cognitive Neuroscience and Learning and IDG/McGovern Institute for Brain Research, Beijing Normal University, Beijing, China; ^2^Center for Cognition and Neuroergonomics, Beijing Normal University, Zhuhai, China; ^3^School of Systems Science, Beijing Normal University, Beijing, China

**Keywords:** habitual exercise, working memory precision, working memory storage capacity, contralateral delay activity, retrospective cue

## Abstract

**Introduction:**

Working memory (WM) is a well-known fundamental ability related to various high-level cognitive functions, such as executive functioning, decision-making, and problem-solving. Although previous studies have posited that chronic exercise may improve cognitive functions, its underlying neural mechanisms and whether habitual exercise is associated with individual WM ability remain unclear.

**Methods:**

In the current study, 36 participants reported their habitual physical activity through the International Physical Activity Questionnaire (IPAQ). In addition to assessments of intelligence quotient (IQ), WM storage capacity (K score), and visuomotor coordination capacity, electroencephalogram (EEG) signals were recorded while the participants performed a WM precision task fusing conventional visual and motor retrospective cue (retro-cue) WM tasks.

**Results:**

We found that greater amounts of and higher frequencies of vigorous-intensity exercise were highly correlated with smaller recall errors in the WM precision task. Contralateral delay activity (CDA), a well-known WM-related event-related potential (ERP) component evoked by the valid retro-cue, predicted individual behavioral recall error. Participants who met the medium or high level of IPAQ criteria (the regular exercise group) showed smaller behavioral recall error and larger CDA than participants who did not meet the criteria (the irregular exercise group). The two groups did not differ in other assessments, such as IQ, WM storage capacity, and visuomotor coordination ability.

**Discussion:**

Habitual exercise was specifically correlated with individual differences in WM precision, rather than IQ, WM storage capacity, and visuomotor coordination ability, suggesting potential mechanisms of how modulations of chronic exercise improve cognition through visual and/or motor WM precision.

## 1. Introduction

Working memory (WM) is a limited-capacity cognitive system that stores and processes information temporarily to sustain complex thought processes and serve various upcoming cognitive tasks and actions (Baddeley, 2012). Previous studies have shown that WM ability is the basis for various advanced and complex cognitive processes in humans, such as fluid intelligence (Johnson et al., 2013; Luck and Vogel, 2013), decision-making (Jimura et al., 2018), and problem-solving (Huang et al., 2014), and that WM training could improve overall cognitive ability and fluid intelligence (Jaeggi et al., 2008; Klingberg, 2010; Karbach and Verhaeghen, 2014). However, WM ability varies greatly from person to person (Luck and Vogel, 2013; Sarigiannidis et al., 2016). It is difficult to improve individual WM ability through behavioral training or electrical stimulation (Melby-Lervåg and Hulme, 2016; Nilsson et al., 2017; Spencer-Smith et al., 2020; Sloan et al., 2021). Acute and chronic exercise have been found to enhance WM task performance (Padilla et al., 2014; Dodwell et al., 2019), and habitual physical activity (PA) has been found to be associated with WM task performance (Eyme et al., 2019). Therefore, individual differences in habitual physical activity might account for individual differences in WM capacity.

The beneficial effects of habitual physical activity on cognitive functions are well established (Chang et al., 2013; Voelcker-Rehage and Niemann, 2013; Giles et al., 2017). This establishment has been built mostly on the positive effects of exercise on brain and cognitive performance in the population of older adults (Erickson et al., 2011; Etnier et al., 2019; Eyme et al., 2019) and children and adolescents (Biddle et al., 2019; Heinze et al., 2021; Ludyga et al., 2022). Compared with older adults, children, and adolescents, whose brains are in decline or still developing, young adults show smaller changes in brain development during training or intervention (Prakash et al., 2015; Ludyga et al., 2020; Nakagawa et al., 2020). The beneficial effects have been less explored in healthy young adults (Guiney and Machado, 2013; Lubans et al., 2016). However, the association between habitual physical activity and better cognitive performance interacts with age (Ludyga et al., 2020). For example, Hillman et al. (2006) found that the association between increased physical activity and higher accuracy in the incongruent flanker task was only significant in the older cohort. Moret et al. (2022) found positive effect of exergame training (physical exercise combined with a videogame) on executive control in older adults whereas the effect is not significant in healthy young adults (Moret et al., 2021). Therefore, it is important to investigate beneficial physical activity effects in the population of healthy young adults (Prakash et al., 2015; Ludyga et al., 2020).

In addition, investigation of healthy young adults is especially important for resolving the debate on whether beneficial exercise effects are general or cognitive domain specific. Chronic exercise has a broad influence on various cognitive functions, such as executive functions, attention, and inhibition (Jonasson et al., 2017; Etnier et al., 2020; Nakagawa et al., 2020), and whether the benefits are general or specific to a narrow range of functions has long been of interest. Through systematic review and meta-analysis, Ludyga et al. (2020) found that the effect size of long-term exercise on complex attention, executive function and memory do not differ significantly, supporting the general rather than cognitive domain-specific exercise effect. However, the differential effects of various exercise trainings and the differential changes in different brain regions suggest domain-specific effects of exercise on cognition (Voelcker-Rehage and Niemann, 2013; Jochem et al., 2017; Rehfeld et al., 2018). The specificity of older adults might reconcile these results. Compared with younger adults, older adults recruit more of the prefrontal cortex to perform various kinds of tasks (Reuter-Lorenz and Cappell, 2008), such as location matching (Grady et al., 1994), working memory, visual attention, and episodic retrieval tasks (Cabeza et al., 2004). The prefrontal cortex of older adults is widely found to be enhanced by physical activity (Jochem et al., 2017; Etnier et al., 2020; Ludyga et al., 2020). Therefore, it is not surprising to find support for the general effect in older adults. The investigation of healthy young adults could contribute to comprehensively answering the long debate about general or specific exercise effects.

In regard to the debate on general exercise benefits or special exercise benefits, it is important to distinguish various cognitive processes, whereas it is difficult to do so through complex behavioral tasks. Few studies have found an association between engagement in habitual physical activity and better behavioral performance in cognitive tasks in healthy young adults (Hillman et al., 2006). For example, college students who meet the physical activity requirements specified by the Centers for Disease Control and Prevention demonstrate better performance in a reading span WM task than their physically inactive counterparts do (Lambourne, 2006). Felez-Nobrega et al. (2017) used participants’ performance in three types of WM complex span tasks (CSTs) as participants’ WM ability and found a positive association between moderate-intensity physical activity and WM ability. However, better performance in WM tasks does not necessarily mean higher WM ability. For example, in the widely used WM complex span tasks, participants need to maintain items in WM while performing distracting cognitive operations and to recall in the correct order of remembered items. The CSTs do not just involve the temporal maintenance and manipulation of information in the brain (Jonasson et al., 2017; Etnier et al., 2020); they also involve multiple cognitive processes in the distracting task. For example, it involves math operations in Operation span CSTs tasks. Because of the multiple processes involved in the WM tasks, it is hard to distinguish WM ability with behavioral measurements in one task. Unknown is the extent to which the positive association between physical activity and behavioral performance in WM complex span tasks reflects the correlation between physical activity and WM ability.

In the last 2 decades, studies have established refined paradigms to probe visual WM ability and have found neural markers of visual WM ability through these tasks (Vogel and Machizawa, 2004; Bays and Husain, 2008; Luck and Vogel, 2013). Visual WM ability has been widely referred to as WM storage capacity, the maximal number of items one could maintain in visual WM at a time (Luck and Vogel, 2013; Zhao et al., 2022), and it is behaviorally widely indexed by the K score in the change-detection task (Pashler, 1988; Cowan, 2001; Vogel and Machizawa, 2004; Zhang and Luck, 2011). In the change-detection task, participants are asked to memorize the visual features (such as colors or orientations) of several items during the encoding period; the items disappear during the retention period. Participants are then asked to report whether any of the items change when items reappear during the probe period. Johnson et al. (2013) measured participants’ K score with the change-detection task and found that it correlated robustly and closely with higher-order cognitive ability measured with the Wechsler Adult Intelligence Scale (WAIS) and MATRICS Consensus Cognitive Battery. Moreover, abundant studies found that the sustained contralateral delay activity (CDA) during the retention intervals is the electrophysiological marker of visual WM capacity: The amplitude of CDA tracks the number of items stored in visual WM and it asymptotes when the stored items reach the individual storage capacity (Vogel and Machizawa, 2004; McCollough et al., 2007).

In addition to storage capacity, emerging studies point to another essential feature of visual WM, precision. Precision is the quality and flexibility of WM, and details of object representations are maintained in visual WM with precision (Bays and Husain, 2008; Ma et al., 2014). Based on the continuous resource model (Bays and Husain, 2008; Bays et al., 2009), visual WM is considered a continuous resource that can be flexibly allocated to all items. Although the volume of this resource is limited, there is no limit on the number of storable items. Therefore, the precision of each item varies, depending on how much resource the brain allocates to it. The amplitude of CDA can also track individual visual WM precision (Fukuda et al., 2010; Göddertz et al., 2018; Machizawa et al., 2020). Specifically, the CDA amplitude in correctly recalled trials is larger than that in incorrectly recalled trials (McCollough et al., 2007) or larger in trials that need fine recall than in trials that need coarse recall (Machizawa et al., 2012, 2020). The quantity-related storage capability and quality-related precision of visual WM seem to be separate and dissociable components of visual WM (Xu and Chun, 2006; Machizawa and Driver, 2011; Ku et al., 2015; Xie and Zhang, 2017).

Habitual physical activity is potentially associated with WM ability since brain structures and functions that are associated with physical activity are associated with individual differences in WM ability (Luck and Vogel, 2013; Voss et al., 2013; Ma et al., 2014; Köhncke et al., 2018). For example, increased brain gray matter volume in the parietal–frontal network of healthy older adults was associated with higher amounts of exercise per week (Eyme et al., 2019). After 4 months of exercise, overweight children demonstrated improvements in executive functions and achievement accompanied by altered parietal–frontal activation in antisaccade tasks (Davis et al., 2011). A comparative study between macaques and humans suggests the frontal activity contributions to the surface activity CDA (Reinhart et al., 2012). Moreover, gray matter volume in the right inferior parietal sulcus (IPS) was associated with individual differences in visual WM precision in terms of both behavioral performance and CDA (Machizawa et al., 2020). All above evidence together suggests that there might be a close correlation between habitual exercise and individual differences in WM ability.

Together, although the beneficial effects of habitual physical activity are widely advocated, whether the effects are general or cognitive domain specific is unclear, and how much physical activity accounts for individual differences in WM ability remains unknown. In the present study, we assessed visual WM ability by electrophysiological marker CDA, behavioral quality-related precision, and behavioral quantity-related storage capacity K score and quantified the association between visual WM ability and habitual exercise. Based on previous findings that chronic exercise could improve performance in multiple WM tasks and that gray matter volume in the exercise-associated parietal–frontal network is associated with individual differences in WM precision, we hypothesized that there is a close relationship between habitual exercise and the amplitude of CDA. We attempted to further explore which subcomponent of WM, storage capacity or precision, relates more to habitual exercise.

## 2. Materials and methods

### 2.1. Participants and study design

Participants were recruited through online advertisements on the Bulletin Board System of Beijing Normal University (BNU). Participants were undergraduate and graduate students of BNU. We used power analysis with G*power software (Faul et al., 2009) to estimate an adequate sample size for our study. Based on effect size in the most relevant study to ours (Lambourne, 2006), the algorithm generated a suggested size of 15 for regular exercise group and 13 for irregular exercise group (please see details about what parameters were used for the estimation in Supplementary material).

Thirty-six healthy young adults (18 females; age range: 19–28 years; mean age: 23.2 ± 2.50 years) participated in the experiment. All of them had normal or corrected-to-normal vision without color blindness and were right-handed. Participants completed multiple behavioral measures, including an intelligence quotient (IQ) test, visual WM change-detection task, visuomotor task, and habitual physical activity questionnaire. Participants performed the visual WM precision task and their EEG signals were recorded. Four young adults were excluded because there were missing data on the frequency or duration of exercise or the sum of the daily exercise time exceeded 960 min; two additional participants were excluded because they showed excessive artifacts in EEG signals. Ultimately, 30 participants were included. All participants provided written informed consent and were compensated in the amount of 75 RMB (approximately 10 USD) per hour. The study was approved by the Beijing Normal University Institutional Review Board.

### 2.2. Exercise level assessment

The International Physical Activity Questionnaire (IPAQ) is an international well-validated questionnaire that measures physical activities (Craig et al., 2003; Hagströmer et al., 2006). In our study, participants’ physical activity was measured by the Chinese-translated version of the IPAQ-Long (Fan et al., 2014; Gao et al., 2022), which measured the weekly exercise level during the month before the questionnaire. The 27-item IPAQ records the physical activity type (work, transportation, household, and exercise), intensity (walking, moderate, and vigorous), duration (at least 10 min or more per day, min/day), and frequency (number of days per week, d/w), showing a test–retest reliability of 0.66 for physical activity in exercise domain and a concurrent validity of 0.37 for moderate and vigorous physical activity in assessing physical activity among Chinese college students (Gao et al., 2022). In the present study, only the measures of exercise were included in the further analysis since all the participants lived on campus and did not show much of physical activity at work, *via* transportation or at home (dormitory). These three types of physical activity were included in the questionnaire so that participants would not combine them with exercise and not over report exercise measures.

The amount or dose of exercise at a certain intensity is the weighting duration (min/d) and frequency (d/w) of that exercise by its energy requirements defined in METs to yield a score in MET-minutes/week. Metabolic equivalents of task (METs) of exercise at a certain intensity are multiples of the resting metabolic rate when that exercise is performance; according to guidelines for IPAQ data analysis (Craig et al., 2003), the METs of vigorous intensity exercise, moderate intensity exercise and walking were assigned to 8, 4, and 3.3 METs, respectively. The total amount of exercise of all intensities was achieved by summing the amount of exercise at all intensities.

The participants were divided into regular and irregular exercise groups based on their levels of habitual exercise according to IPAQ categorization criteria. Participants were categorized into the regular exercise group if their exercise level met the medium or high level of the IPAQ categorization criteria, which includes: (a) 3 or more days of vigorous-intensity exercise of at least 20 min/d; (b) 5 or more days of moderate-intensity exercise and/or walking at least 30 min/d; or (c) 5 or more days of a combination of three intensities exercise achieving at least 600 MET-min/w. Participants who met the low level of the IPAQ categorization criteria were categorized into the irregular exercise group. Fifteen participants (nine females; age range: 20–28 years; mean age: 22.6 ± 2.26) were grouped into the regular exercise group, and the other 15 participants (eight females; age range: 19–27 years; mean age: 23.9 ± 2.70) were grouped into the irregular exercise group. Notably, regular engagement is an important concept included in current public health guidelines for physical activity and lifestyle (Haskell et al., 2007; O’Donovan et al., 2010). Therefore, IPAQ categorization criteria emphasize not only the total amount of exercise but also the frequency and duration within each day.

### 2.3. Retro-cue WM precision task

Visual WM precision was measured with a WM task with a retro-cue fusing traditional visual and motor WM tasks. Participants’ EEG signals were recorded simultaneously when they performed the retro-cue task to obtain ERP component CDA.

#### 2.3.1. Stimuli and procedure

**Figure 1:**
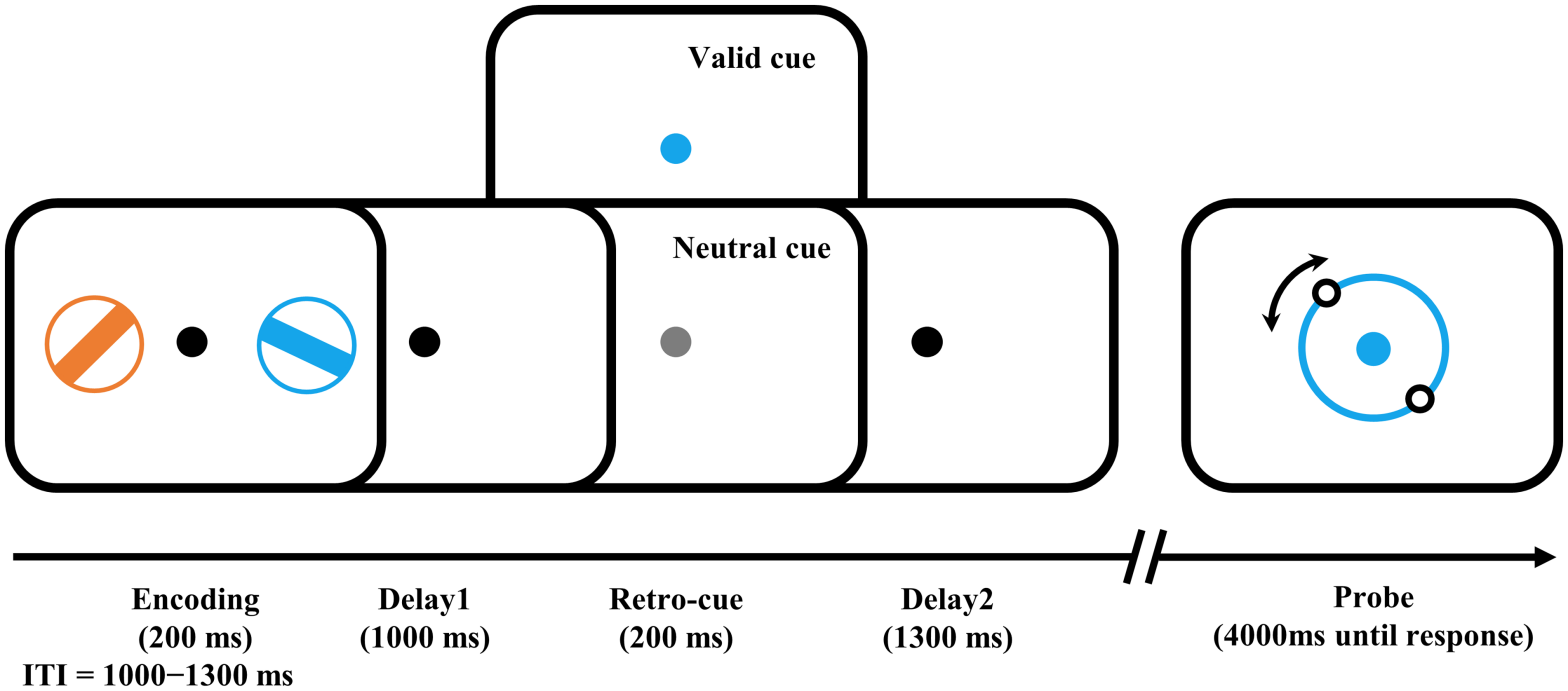
Examples of trial schedule and experimental design. The number of valid cues and neutral cues were equal. Participants needed to rotate the illusory orientation of the two dots on the probe ring to the orientation of the target bar, i.e., the bar in the encoding phase with the same color as the probe ring. ITI, intertrial interval.

The visuomotor WM task (Figure 1) was presented with PsychoToolbox in MATLAB. The stimuli were presented on a 21-in LCD monitor (800 × 600 pixels, 60 Hz) with a viewing distance of 80 cm. During the task, a central fixation spot (0.5°) was presented on a gray background. At the encoding stage, two different colored bars (4° × 0.32°) with different orientations surrounded by a ring (0.32° thick) were presented in each visual field (5° to the left and right of the black fixation) for 200 ms. The colors of the two bars were randomly and exclusively chosen from blue (RGB: 21, 165, 234), orange (RGB: 234, 74, 21), green (RGB: 133, 194, 18), and purple (RGB: 197, 21, 234). Their orientations were also randomly and exclusively selected from ±11.25, ±33.75, ±56.25, to ± 78.75°, resulting in a 22.5° or more difference between the two bars in each trial. After a delay of 1,000 ms, the retro-cue was presented with the color-changed central fixation spot for 200 ms. In half of the trials, the color of the cue was randomly changed to either color of the two bars as a valid cue to indicate which bar would be the target bar needed to recall in that trial. In the other half of the trials, the color was changed to light gray as a neutral cue to indicate that either bar would be the target. Following another 1,300 ms delay after the retro-cue offset, a probe was displayed in the color of the target bar, that is, the cued bar in valid-cue trials or the color of one of the two bars presented in the encoding stage in neutral-cue trials. Participants were instructed to rotate the probe bar’s orientation using a computer mouse and provide a click response when it matched the target orientation. The goal was to be as precise as possible within a 4,000 ms timeframe with precision emphasized over response time. This mouse report was a continuous report, ranging from 0 to 360°. The intertrial interval (ITI) varied randomly between 1,000 and 1,300 ms.

The formal experiment was divided into 20 blocks of 56 trials each with a 1-min break between blocks, and a block of trials was performed before the formal experiment to help participants become familiar with the task. The recall errors, i.e., the angular difference between the reported and the target bar’s orientation, and the response times (RTs), i.e., the time from probe presentation to the mouse click, were recorded for further analysis.

#### 2.3.2. EEG recording and analysis

The participants’ EEG activities were recorded using a cap with 64 AgCl electrodes in accordance with the international 10–20 system and a SynAmps EEG amplifier (NeuroScan, Inc.), while performing the retro-cue WM task. Eye movements and blinks were monitored using two electrodes placed 1 cm above and below the left eye for vertical electrooculogram and another two electrodes placed at the outer canthus to the eyes for horizontal electrooculogram. Before recording, electrode impedance was maintained below 5 kΩ. Signals were referenced online to the left mastoid, amplified with a bandpass of 0.01–400 Hz, and digitized online at a sampling rate of 1,000 Hz.

The EEGLAB toolbox in MATLAB was used to process the collected data. The data were downsampled to 256 Hz, filtered with a bandpass of 0.1–40 Hz, and rereferenced offline to the average of two mastoids. Relative to retro-cue onset, epochs were extracted from −200 to 2,000 ms. Eyeblink and movement artifacts were corrected by independent component analysis (ICA). There were 6.867 ± 5.063 ICA components removed on average. After ICA, the baseline correction was performed from −200 ms to retro-cue onset in each epoch. The epochs in which there were voltages exceeding ±80 μV or any left artifacts detected by the eye were excluded. There were 2.38% of the epochs on average (range: 0–10.54%) were removed, resulting in 547 ± 16 epochs for the valid condition and 546 ± 17 epochs for the neutral condition.

To measure the CDA component, we determined the time window and electrodes basing on previous studies with similar paradigm (Göddertz et al., 2018; Li et al., 2023). we calculated the contralateral and ipsilateral waveforms relative to the target-located hemifield by averaging across five pairs of electrode sites (P3/4, P5/6, P7/8, PO5/6, and PO7/8). That is, waveforms from P4, P6, P8, PO6, and PO8 were averaged as the contralateral waveform and waveforms from P3, P6, P7, PO5, and PO7 were averaged as the ipsilateral waveform when the target was presented in the left visual field and vice versa when the target was presented in the right visual field. Finally, both the contralateral and ipsilateral waveforms were averaged across trials and target-located hemifield for each cue condition and each participant. To calculate the amplitude of CDA, a difference wave was created by subtracting the ipsilateral waveforms from the contralateral waveforms, and the average voltage across the time window of 800–1,300 ms after retro-cue onset was calculated as the amplitude of CDA. The exact time points at which the CDA was significant were 762–1,285 ms (please see details about how we found these points in Supplementary material).

To get reliable results, the experiment includes 1,120 trials in total, which might lead to learning effect or fatigue effect at the end. We checked these effects by comparing the indexes in the first and second halves of the trials (see details in Supplementary material).

### 2.4. Behavioral measures

To assess participants’ general intellectual ability, their IQ was measured by the WAIS-III (Wechsler, 1997). Visual WM quantity-related storage capacity was measured through a widely used change detection paradigm in which participants reported whether the probe items were the same as the items that appeared before (Vogel and Machizawa, 2004; see details in Supplementary material), and its behavioral index K score was calculated by Cowan’s formula (Cowan, 2001). To check whether the potential correlation between habitual exercise and visuomotor WM precision was mediated by basic visuomotor coordination, we measured participants’ basic visuomotor coordination performance. Visuomotor coordination was measured through a widely used paradigm, in which participants were asked to rotate the direction of the probe bar to the direction of the target bar, which was presented on the screen throughout the task (Burnett Heyes et al., 2012; see details in Supplementary material).

### 2.5. Statistical analysis

All statistical analyses were performed using SPSS version 26. To compare the differences between the two exercise groups, the independent-sample *t* test, the chi-square test, and the Mann–Whitney *U* test were used to analyze different types of data. The chi-square test was used on gender distribution; the Mann–Whitney *U* tests were used for exercise measures (the amount and frequency of exercise); the independent-sample *t* tests were used for the other demographic variables except gender and behavioral assessments. Two-way repeated-measures ANOVA was used with the between-subject factor for Group (regular exercise, irregular exercise) and the within-subject factor for Retro-cue type (valid cue, neutral cue). The one-sample *t* test was used to check for existence of CDA components, and Spearman’s correlation analysis was used to test the relationship between two variables. The statistical significance level was set at 0.05 for all analyses. The effect size Cohen’s *d* scores for the *t* test were calculated using jamovi.

## 3. Results

Demographic information, behavioral assessments, and exercise variables for the two groups are shown in Table 1. The two exercise groups did not differ in gender (χ^2^ = 0.136, *p* = 0.713), age, and education (*t*s ≤ 1.394, *p*s ≥ 0.174), suggesting the two samples were homogenous. The regular exercise group differed from the irregular exercise group only in habitual exercise (see details below), not in IQ, K score, or visuomotor coordination (*t*s ≤ 1.677, *p*s ≥ 0.107).

**Table 1:** Demographic information, cognitive abilities, and exercise variables of the two groups.

Variables	Irregular exercise group	Regular exercise group	Group difference
Gender (male/female)	6/9	7/8	χ^2^ = 0.136
Age (years)	23.87 ± 2.696	22.60 ± 2.261	*t* = 1.394
Education (years)	17.93 ± 1.870	17.15 ± 1.463	*t* = 1.214
IQ	128.47 ± 4.406	130.77 ± 5.659	*t* = 1.210
K scores	1.92 ± 0.774	2.26 ± 0.493	*t* = 1.352
Visuomotor precision (°)	5.10 ± 1.341	4.20 ± 1.344	*t* = 1.677
Amount of exercise (MET-min/w)			
Vigorous	0.00 (0.000)	600.00 (1400.000)	*U* = 41.000^**^
Moderate	0.00 (0.000)	320.00 (540.000)	*U* = 42.500^**^
Walking	198.00 (181.500)	462.00 (379.500)	*U* = 69.500
Total	231.00 (377.500)	1253.00 (2212.500)	*U* = 18.000^***^
Frequency of exercise (d/w)			
Vigorous	0.00 (0.000)	3.00 (3.000)	*U* = 34.000^***^
Moderate	0.00 (0.000)	1.00 (4.000)	*U* = 51.500^**^
Walking	2.00 (1.000)	7.00 (3.000)	*U* = 61.000^*^

Values are M ± SD or Median (Interquartile Range). Statistical test values represent the differences between the regular exercise and irregular exercise group. MET, metabolic equivalent; min/w, minutes per week; d/w, days per week; ^*^p < 0.050, ^**^p < 0.010, and ^***^p < 0.001.

### 3.1. Exercise reports

The regular exercise group demonstrated significantly more amounts of and higher frequencies of vigorous and moderately intense exercise than the irregular exercise group (*U*s ≤ 51.500, *p*s ≤ 0.006). Although the two groups showed a significant difference in the frequency of walking (*U* = 61.000, *p* = 0.027), they did not show a significant difference in the amount of walking (*U* = 69.500, *p* = 0.073), resulting in a greater difference in the total amount of exercise (*U* = 18.000, *p* < 0.001). These observations confirmed the effectiveness of our approach of allocating participants into groups in terms of their habitual exercise level.

### 3.2. Behavioral and ERP results in the retro-cue WM task

#### 3.2.1. Behavioral results

**Figure 2:**
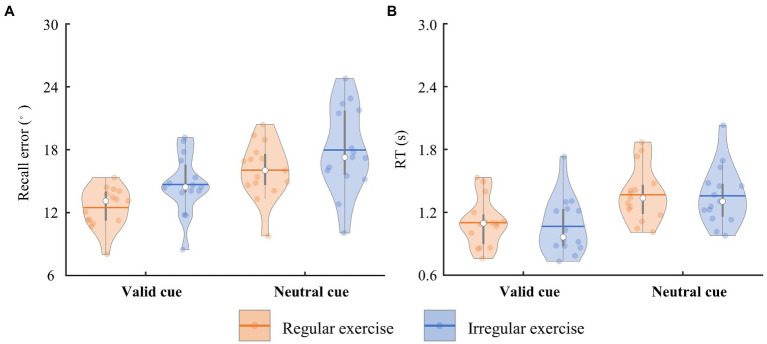
The trial-averaged recall error and RT of the two groups. **(A)** The recall error of the regular and irregular exercise groups in the neutral and valid cue conditions. The horizontal bars indicate the averaged values across participants. Each dot marks a participant. Same below. **(B)** The RT of the regular and irregular exercise groups in the neutral and valid cue conditions.

The participants reported the orientation of the target bar in 0.733–2.031 s (referred to as RTs) on average in the task. The error between the reporting orientation and the target bar orientation (referred to as recall error) varied from 8.012 to 24.774° on average. To quantify the differences between the regular exercise group and irregular exercise group, mixed ANOVA with retro-cue type and group was performed on participants’ mean recall errors and RTs. Participants in the regular exercise group showed better performance in the WM task, supported by a significant main effect of group on recall errors [*F*(1, 28) = 4.274, *p* = 0.048, η_p_^2^ = 0.132; Figure 2A]. Performance in the valid trials was more precise equally in two groups, supported by a significant main retro-cue effect [*F*(1, 28) = 72.303, *p* < 0.001, η_p_^2^ = 0.721] and by a nonsignificant interaction effect [*F*(1, 28) = 0.117, *p* = 0.735, η_p_^2^ = 0.004]. No significant group effect was found in RTs [*F*(1,28) = 0.060, *p* = 0.808, η_p_^2^ = 0.002], neither the interaction between group and retro-cue [*F*(1, 28) = 0.659, *p* = 0.424, η_p_^2^ = 0.023]. Only the main effect of retro-cue was observed on RTs [*F*(1, 28) = 308.219, *p* < 0.001, η_p_^2^ = 0.917; Figure 2B].

#### 3.2.2. WM-related CDA components

**Figure 3:**
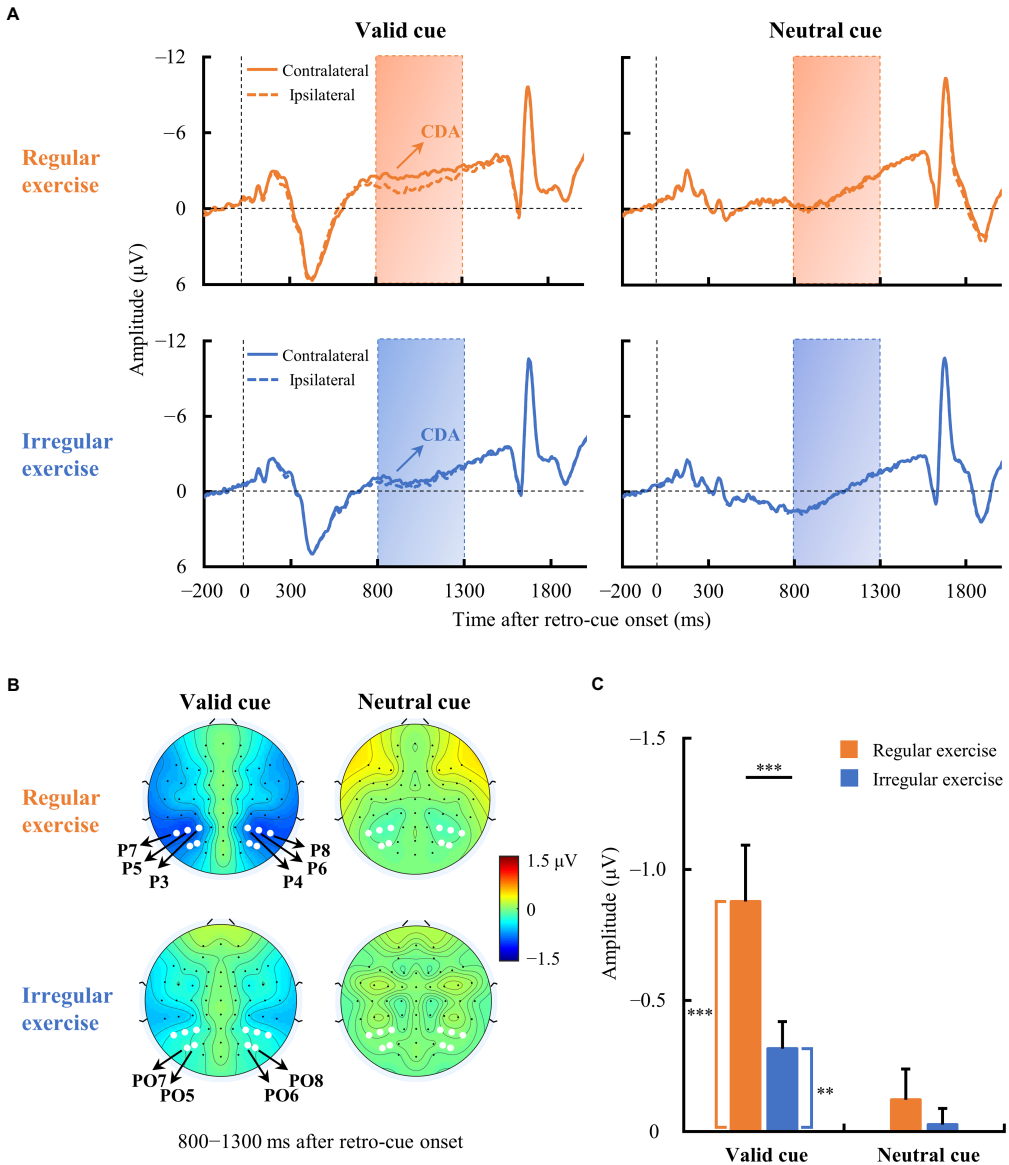
Event-related potential (ERP) waveforms averaged across contralateral and ipsilateral electrodes in the two exercise groups. **(A)** Averaged ERPs at contralateral and ipsilateral electrodes of the regular and irregular exercise groups in neutral and valid cue conditions. The horizontal dotted line indicates 0 μV, and the vertical dotted line indicates the time of retro-cue onset. The rectangular area is the time window (800–1,300 ms) for CDA amplitude calculation. **(B)** Topographic maps of contralateral-minus-ipsilateral difference waves (800–1,300 ms after retro-cue onset) of regular and irregular exercise groups in neutral and valid cue conditions. The white dots are the electrodes (P3/4, P5/6, P7/8, PO5/6, and PO7/8) for ERP analysis. **(C)** Mean amplitudes of the difference waves between 800 and 1,300 ms after retro-cue onset of regular and irregular exercise groups in neutral and valid cue conditions. Error bars represent standard errors of the mean. ^**^p < 0.010, ^***^p < 0.001.

Figure 3A illustrates the averaged ERP waveforms evoked by the retro-cue in the regular and irregular exercise groups from contralateral and ipsilateral electrodes over the parieto-occipital scalp. The regular exercise group demonstrated clear traditional CDA in the valid cue condition, i.e., the contralateral activity within 800–1,300 ms of the ERP waveform was more negative than the ipsilateral activity (Figure 3B). The amplitude of CDA seemed to be smaller in the valid cue condition in the irregular exercise group and it was absent in the neutral cue condition in both groups. To quantify these ERP differences between contralateral and ipsilateral waves, we measured the amplitude of CDA by the mean voltage within 800–1,300 ms in the contralateral-minus-ipsilateral difference waves. To test whether CDA exists, one-sample *t* tests were performed on CDA amplitudes against zero in each condition and each group (Figure 3C). The results suggest robust CDA in the valid cue condition in both the regular exercise group [−0.878 ± 0.830 μV; *t*(14) = −4.095, *p* < 0.001, Cohen’s *d* = 1.057] and the irregular exercise group [−0.316 ± 0.400 μV; *t*(14) = −3.064, *p* = 0.008, Cohen’s *d* = 0.791]. Further independent sample *t* tests suggested a much larger CDA in the regular exercise group than in the irregular exercise group [*t*(28) = 2.361, *p* = 0.025, Cohen’s *d* = 0.862]. No reliable CDA was presented in the neutral cue condition either in the regular exercise group [−0.121 ± 0.458 μV; *t*(14) = −1.022, *p* = 0.324, Cohen’s *d* = 0.264] or in the irregular exercise group [−0.027 ± 0.236 μV; *t*(14) = −0.439, *p* = 0. 667, Cohen’s *d* = 0.113]. Note that the color of the cue in the neutral condition was gray, meaning that the target would be presented in either visual field. The target was not defined until the probe was presented. It was reasonable to find no retro-cue evoked CDA in the neutral condition since participants did not know which field of item would be retrieved during the retro-cue period.

**Figure 4:**
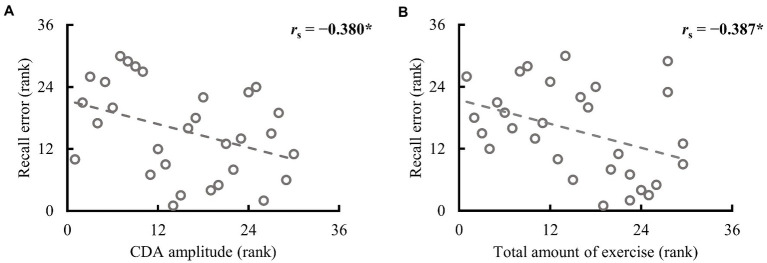
Spearman correlation between recall error in the valid cue condition and CDA amplitude and the total amount of exercise in the full sample (*n* = 30). **(A)** The higher the rank is, the larger the CDA amplitude and recall error. **(B)** The higher the rank, the larger the total amount of exercise and recall error. ^*^p < 0.050.

Participants in both groups demonstrated clear CDA in the valid cue condition, and the two groups differed significantly (see Figure 3C). To further explore whether CDA tracks individual differences in WM precision, Spearman correlation analysis was performed between CDA amplitudes and recall errors in the valid cue condition across all participants. As illustrated in Figure 4, there was a significant negative correlation between CDA amplitudes and recall errors (*r_s_* = −0.380, *p* = 0.038), indicating that WM precision was higher in participants with greater CDA. The amplitudes of CDA evoked by the valid retro-cue was also significantly correlated with K score (*r*_s_ = 0.393, *p* = 0.039), confirming the role of CDA in tracking individual WM storage capacity.

### 3.3. Correlation between habitual exercise and WM precision

To determine which variables in habitual exercise significantly contributed to group differences in performance in the retro-cue task, Spearman correlation analyses were used to explore the relationship between the amount and frequency of different intensities of exercise and WM precision across all participants. The results are demonstrated in Table 2. As illustrated in Figure 4B, a significant correlation was found between recall error in the valid cue condition and the total amount of exercise (*r_s_* = −0.387, *p* = 0.035). Specifically, recall errors were correlated with vigorous-intensity exercise (amount: *r_s_* = −0.437, *p* = 0.016; frequency: *r_s_* = −0.483, *p* = 0.007); no significant correlations were observed between recall errors and walking exercise (*r_s_*s ≤ 0.174, *p*s ≥ 0.292). These observations suggest specific correlations between recall error in the valid cue condition and vigorous intensity exercise. No significant correlations were observed between CDA amplitudes and the total amount of exercise (*r_s_* = 0.237, *p* = 0.208) and the amount of vigorous-intensity exercise (*r_s_* = 0.257, *p* = 0.170). Caution is needed in interpreting these correlations alone because of the small sample size of this study, however, the results are still considered to be reliable (see Discussion and Supplementary material for details).

**Table 2:** Spearman correlation between exercise and recall errors in the full sample (*n* = 30).

Amount and frequency of exercise	Recall error (rank)	
	Valid cue	Neutral cue
Amount of exercise (rank)		
Vigorous	−0.437^*^	−0.238
Moderate	−0.037	−0.149
Walking	−0.199	−0.066
Frequency of exercise (rank)		
Vigorous	−0.483^**^	−0.347
Moderate	−0.019	−0.195
Walking	−0.016	0.174

^*^p < 0.050 and ^**^p < 0.010.

## 4. Discussion

Working memory ability is a fundamental ability of several high-level cognitive functions and actions in daily life, fluctuating substantially from person to person. In the present study, we explored whether habitual exercise could explain individual differences in different subcomponents of WM ability (capacity and precision). In a retro-cue task while their EEG signals were being recorded, participants with various amounts and frequencies of habitual exercise reported the precise orientation of retained items in their WM with a mouse. Their IQ, visual WM storage ability and basic visuomotor coordination were assessed outside the retro-cue experiment. Participants’ habitual exercise was measured through IPAQ, and they were categorized into the regular or irregular exercise group according to whether their level of exercise was above the low level of IPAQ categorization criteria. We found that individuals with regular exercise demonstrated a better WM precision and a larger WM-related ERP component CDA compared with their counterparts with irregular exercise.

### 4.1. Habitual exercise was not only related to better behavioral WM performance, but also to larger CDA

In line with previous studies (Lambourne, 2006; Felez-Nobrega et al., 2017), we found that greater amounts of and higher frequencies of habitual vigorous exercise were correlated with better performance in the retro-cue WM precision tasks. Consistently, participants who performed regular exercise showed better WM precision. Specifically, the CDA was larger in the regular exercise group than in the irregular exercise group, suggesting that better behavioral performance associated with habitual exercise are related to better WM ability, not just WM task performance.

### 4.2. The relationship was specific to WM precision

The better behavioral performance in the regular exercise group in our study could be at least ruled out from group differences in brain functions, including attention, IQ, WM storage capacity, and visuomotor coordination. (1) The retro-cue effect (difference between the valid and neutral conditions) reflects attentional modulation of the retro-cue (Souza and Oberauer, 2016). The nonsignificant interaction effect between group and cue in the retro-cue task indicates a nonsignificant attentional modulation difference between the two groups. (2) The two groups did not differ in IQ, WM storage capacity K score, or visuomotor coordination. All these measures except WM precision were not significantly correlated with habitual exercise. Together, these results suggest that habitual vigorous exercise is more closely related to individual differences in WM precision.

### 4.3. The relevant characteristics of exercise: Intensity, frequency, and amount

In the present study, WM precision was not correlated with all intensities of habitual exercise but was specifically correlated with vigorous exercise. This is consistent with the association between stronger self-reported intensity of PA and better working performance found previously (Köhncke et al., 2018) and with the findings that only moderate to vigorous exercise training is effective on improving cognition (Dodwell et al., 2019; Nakagawa et al., 2020). However, the intensity effect interacts with the term of exercise (Roig et al., 2013). For example, Dodwell and colleagues found benefits of moderate acute exercise on CDA (Dodwell et al., 2019) and on attentional processing (Dodwell et al., 2021), whereas no effect of vigorous acute exercise was observed (Dodwell et al., 2021). Exercise performed immediately prior to a visual perceptual learning task even impairs learning (Connell et al., 2018). The effect of the intensity of exercise on exercise benefits also interacts with age (Prakash et al., 2015; Ludyga et al., 2020). For example, walking is effective for older adults, and only moderate to vigorous walking is effective for young adults (Nakagawa et al., 2020). In general, superior effects are found in older adults, and it takes engagements in exercise of a stronger intensity to have an effect on young adults (Haskell et al., 2007; O’Donovan et al., 2010).

The present study found that both the frequency and amount of vigorous exercise were related to WM precision. The dose (amount)-response relationship between exercise and cognition has received substantial attention since it is also important for optimizing exercise intervention benefits and the general guideline for habitual physical activity. The amount of a certain exercise is multiplied by its duration and frequency. Studies found the best effect of moderate duration (Colcombe and Kramer, 2003; Northey et al., 2018), which means shorter duration is not enough and prolonged duration is harmful. Compared with “one fits all,” current public health recommends regular engagement in physical activity, which comes from the finding of the importance of frequency (Prakash et al., 2015). Our finding of the correlation between frequency of vigorous exercise and WM precision suggest that frequency or regular engagement is a more relevant factor once the amount of each bout of exercise (intensity, duration) meets the criterion.

### 4.4. Potential explanations for the specific relationship between habitual exercise and WM precision

Habitual exercise was specifically correlated with behavioral recall error in the retro-cue continuous report task, rather than the K score in the change-detection WM storage task, indicating that the beneficial effects on cognition from habitual exercise may result more from improvements in WM precision than from WM storage capacity. These relatively greater benefits on precision could be explained by the following: First, precision is more related to functions in the parietal–frontal network. Machizawa et al. (2020) found that individual WM storage capacity was correlated with gray matter volume in the left lateral occipital cortex (LOC), while precision was correlated with gray matter volume in the right IPS. Visual cortex seems to be less influenced by exercise training since visual perceptual learning performance, and the well-established paradigm of visual plasticity, is not affected by exercise training (Campana et al., 2020). On the contrary, parietal cortex has been consistently observed to be influenced by chronic exercise (Etnier et al., 2019). Second, the WM task used in the present study is a task fusing visual and motor WM tasks, and the report in our task is motor-based, which does not only involve visual but also motor WM. With a similar paradigm, van Ede et al. (2019) found independent and simultaneous visual and motor WM recall after the retro-cue. WM precision in our study is exactly visuomotor WM precision (i.e., the precision in coordination between visual perception and planned movements; Goodale, 1998). Since basic visuomotor coordination behavior was not linked to habitual exercise in our study, the correlation in the retro-cue task were specific to visuomotor WM ability. Besides, exercise facilitate reallocation of neural resources toward networks involved in implicit functions, such as motor control (Dietrich and Audiffren, 2011; Dodwell et al., 2019).The CDA enhanced by acute exercise as well as motor-response selection (Dodwell et al., 2019). The parietal–frontal cortex and basal ganglia have been found to be involved in these functions (Goodale, 1998; Munoz and Everling, 2004) and have changed after chronic exercise (Davis et al., 2011; Heinze et al., 2021). For example, Köhncke et al. (2018) found that self-reported exercise intensity in healthy older adults is associated with the utilization of dopamine in the caudate, which in turn improves memory. In addition, dopamine has also been found to improve WM precision (Zokaei et al., 2012; Blatt et al., 2014; Fallon et al., 2017). Therefore, these results suggest that chronic exercise does not benefit general cognitive capacity but benefits cognition associated with WM precision more. Future investigations on the intervention with exercise could test that.

### 4.5. The marker CDA was less sensitive than behavioral WM precision

As expected, we found that the amplitude of CDA was correlated with behavioral recall errors in the retro-cue task, confirming the role of CDA as the neural marker of WM ability. Surprisingly, the amplitude of CDA did not correlate with habitual exercise, but just differed between the regular and irregular exercise groups, although the behavioral recall error correlated with habitual exercise. The smaller link between CDA and habitual exercise could be due to the following reasons. First, the CDA used to relate to individual differences in WM ability in previous studies is the difference CDA between high load and low load (Vogel and Machizawa, 2004) or between fine representation and coarse representation (Machizawa et al., 2012, 2020), while CDA in our study was the absolute CDA in low load or fine representation. Second, the CDA relates not only to WM precision but also to WM storage capacity, indicating the partial role of CDA in WM precision, while we found habitual exercise was specifically correlated with WM precision rather than WM storage. Fukuda et al. (2016) found that CDA and lateralized alpha modulations represent separate components in WM. Finally, we used a visuomotor retro-cue WM task, which also involves the utilization of the visual WM in addition to retention and recall in the traditional change-detection WM task. van Ede et al. (2019) used the same paradigm and found that visual recall and motor recall started independently and simultaneously. This result might suggest that the amplitude of CDA did not reflect motor WM, which needs to be further explored in future research.

### 4.6. The sample of the present study

The sample size of the present study was 30 and relatively small, which does not represent a larger population. The results from a small sample would not be easily replicated. On the one hand, a small sample would produce false-positive results and inflated effect sizes; on the other hand, it would not be sufficient to detect small effects (Button et al., 2013; Schweizer and Furley, 2016). However, Bacchetti (2013) thought that a small sample size is not the real problem. Our sample size is larger than the estimated sample size by G*Power software; and we argued that our results were reliable. First, the adjusted effect sizes of our main results were still medium to large (see Supplementary Table S1 in Supplementary material for details). Ivarsson et al. (2013) proposed that the adjusted effect size would be better when the sample size is small. We computed the adjusted effect sizes of our main results based on Ezekiel’s formula (see Supplementary material for details; Ezekiel, 1930; Wang and Thompson, 2007). The medium to large adjusted effect size would relieve the potential inflated effect of a small sample on the effect size. In addition, since we focused on the relative sensitivity of WM precision and WM storage capacity to habitual exercise, it would be less likely that the same sample was biased to one of the two components. Therefore, our results of the control variables, such as IQ, attention and visuomotor coordination, would relieve the potential inefficacity of detecting a small effect of a small sample.

The generalization of our results would be limited because of the uniqueness of our sample. Participants in our study were undergraduate and graduate students at our university, a top-ranked university in Mainland China, and they showed a relatively higher than average IQ evaluated by WAIS-III. Since individual differences in WM are closely related to intelligence (Jaeggi et al., 2008; Klingberg, 2010; Johnson et al., 2013), our results might not be applicable to normal healthy young adults. In addition, they lived on campus, not showing much the other domains of physical activity except exercise. A previous study found that sports and exercises are more related to cognitive performance than other types of activity in healthy young adults (Jochem et al., 2017). The correlation might be specific to habitual exercise. Future studies should use a larger and more widely covered sample.

### 4.7. The causation of the relationship

Although correlation does not reflect a causal link and the causation of PA and cognition could be two directions (Köhncke et al., 2018), the uniqueness of our sample might reflect the positive effect of habitual exercise on WM precision more than the other way around. The beneficial effects of physical activity and exercise on cognition have been supported by many long-term intervention studies (Prakash et al., 2015; Etnier et al., 2020). The effect of cognition on physical activity has been supported by the findings that baseline cognitive performance predicted the follow-up physical performance (Tabbarah et al., 2002) and that cognitive functions help to maintain physical activity through subjective energy availability (Cheval et al., 2022). However, physical activity of college students might not be easily influenced by cognitive abilities. For example, Lambourne (2006) found that college students from the Physical Education Department showed more METs and smaller BMIs than students from the Psychology Department, whereas they did not differ in WM task performance. To reduce the effect of cognition on PA, Larson et al. (2006) only included older adults who scored in the top 25% on the Cognitive Ability Screening Instrument. They still found the correlation between PA and cognition, suggesting the causation of the effect of PA on cognition. Similarly, participants in our study showed higher IQ generally, ranging from 117 to 139, which might reduce the potential support for causation from WM precision to habitual exercise.

## 5. Limitations

There are some limitations in the present study. Although we found robust results through different methods (see details in the Supplementary material), the sample size of our study was small. Participants were undergraduate and graduate students with relatively higher IQs and little or no physical activity at work, *via* transportation and at home. Research with larger and more representative samples is needed to verify the specific correlation between habitual exercise and WM precision in future correlation studies. The way we categorize participants was unique, and the label “irregular” should be understood carefully. We categorized individuals into two groups according to whether their exercise was above the low level or not according to IPAQ group categorization criteria. Since the criterion takes intensity, frequency, and amounts into account and emphasizes regular engagement, we label participants whose activity was above a low level regular. Correspondingly, we label individuals whose activity was at a low level irregular. From the grouping criteria, participants who were grouped into the irregular exercise group means they showed an inadequate amount of exercise and not a higher frequency of exercise. Further work is needed to replicate the findings with a large sample size and participants with different levels of intelligence. As cross-sectional research and correlation analysis could not show the causal relationship well, a longitudinal intervention study is needed to examine the causal link between regular exercise and WM precision in the future. Self-reported exercise may be less accurate, although they take individual differences in predispositions into account and likely reflect the degree to which an individual is physically challenged (Köhncke et al., 2018). Objective measures should be considered in the future, such as accelerometers or the use of fitness tests to measure exercise levels. Although event-related alpha desynchronization has always been related to attention (Foster et al., 2016; Souza and Oberauer, 2016), it is also found to be associated with WM ability, which should be further examined (Poliakov et al., 2014; Li et al., 2021).

## 6. Conclusion

The present study found that the amplitude of CDA tracks individual behavioral recall error in the visuomotor retro-cue WM task, with the former distinguishing individuals who engage in regular habitual exercise from those who do not and the latter correlating with the amount and frequency of individual vigorous-intensity habitual exercise. The specific large correlation between habitual vigorous-intensity exercise and WM precision suggests that the beneficial effects of habitual exercise might be through the precision of WM, especially visuomotor precision. These results may spark future investigations on the mediation of visual and/or motor WM precision between cognition and exercise intervention in healthy young adults.

## Data availability statement

The raw data supporting the conclusions of this article will be made available by the authors, without undue reservation.

## Ethics statement

The studies involving human participants were reviewed and approved by the Beijing Normal University Institutional Review Board. The patients/participants provided their written informed consent to participate in this study.

## Author contributions

XY, DL, YH, JH, and YS contributed to conception and design of the study with developed idea from MQ, YK, and CZ. XY, DL, and YH collected the data with the help of MQ and YK. XY, DL, and JH performed the data analysis with constructive discussion from YH, CZ, and YS. XY and DL wrote the manuscript with critical review and editing from JH and YS. All authors contributed to the article and approved the submitted version.

## Funding

The present research was supported by the National Natural Science Foundation of China Grant No. 32271094 to YS, No. 31871099 to YS, and No. 32200870 to JH.

## Conflict of interest

The authors declare that the research was conducted in the absence of any commercial or financial relationships that could be construed as a potential conflict of interest.

## Publisher’s note

All claims expressed in this article are solely those of the authors and do not necessarily represent those of their affiliated organizations, or those of the publisher, the editors and the reviewers. Any product that may be evaluated in this article, or claim that may be made by its manufacturer, is not guaranteed or endorsed by the publisher.
