# Supplementary material for: Neural and behavioral evidence supporting the relationship between habitual exercise and working memory precision in healthy young adults
Source: Front Neurosci. 2023 Apr 6;17:1146465. doi: 10.3389/fnins.2023.1146465 (PMC10116001; doi:10.3389/fnins.2023.1146465)
Supplement: Supplementary file 1 [file Data_Sheet_1.docx]

Supplementary Material

# The estimation of the sample size

The estimation was based on the most relevant paper to our study (Lambourne, 2006). In this study, Lambourne (2006) found that individuals who meet fit requirements (*N* = 23) showed an average working memory performance of 4.5 with an SD of 1.5 and that individuals who did not meet fit requirements (*N* = 19) showed an average performance of 3.2 with an SD of 1.0. Performance of the two groups differ significant (*F* = 9.06, *p* = 0.005, η^2^ = 0.210; see Table 1 of Lambourne, 2006). According to Cohen’s d score calculation, the effect size d score of the two-group difference in that study was 1.0007. Then, with an effect size of 1.0007, alpha level of 0.05, power of 0.8, tail of one, and ratio *N*2/*N*1 of 0.8261 (19/23), we obtained the estimations 15 and 13 for the sample size of the regular exercise group and irregular exercise group, respectively.

# Change detection task

A widely used change detection paradigm (Vogel and Machizawa, 2004) was used to measure participants’ visual WM quantity-related storage capacity. In the task, participants were asked to report whether the probe items were the same as the items that appeared before. At the start of each trial, a memory array consisting of two, four, or six different oriented red bars (2° × 0.14°, RGB: 255, 0, 0) was displayed for 200 ms. Half of them were presented on the left visual hemifield, and the other half was presented on the right. The orientations of the bars were randomly and exclusively chosen from a set of 9 degrees varying from 16° to 176° with a 20° step (16°, 36°, 56°, 76°, 96°, 116°, 136°, 156°, and 176°). Following a delay of 900 ms, a probe bar was presented at one of the memory bars’ locations. Participants were asked to report whether there was an orientation difference between the probe bar and the memory bar at the same location within 3,000 ms. The probability that there was an orientation difference was 50%, in which condition the orientation of the probe bar was chosen from the other 8 degrees randomly. The ITI varied from 1,000 to 1,200 ms. Participants performed 6 blocks and 60 trials each block. The behavioral index K score was calculated by Cowan’s formula (Cowan, 2001) in which *K* = *S* × (*H* - *F*), where *K* is the memory capacity, *S* is the size of the array, *H* is the observed hit rate and *F* is the false alarm rate.

# Visuomotor coordination task

The WM precision task used in the present study is a task fusing visual and motor WM tasks, and the task is to manipulate a mouse to rotate a bar to the target orientation stored in WM. The task is motor-based, which does not only involve visual WM but also motor WM (van Ede et al., 2019). WM precision in our study is exactly visuomotor WM precision (i.e., the precision in coordination between visual perception and planned movements; Goodale, 1998). To check whether the potential correlation between habitual exercise and visuomotor WM precision was mediated by basic visuomotor coordination, we measured participants’ basic visuomotor coordination performance (Burnett Heyes et al., 2012). In each trial, stimuli were the same as that presented in the encoding stage in the retro-cue WM precision continuous report task (Figure 1), whereas a probe was displayed in the center with randomized orientation and with the same color as either of the two bars rather than the black fixation spot. Participants were instructed to match the orientation of the probe with the target (i.e., the bar with the same color as the probe). The two bars were presented on the screen throughout the task, and participants did not need to remember the target orientation. Participants were allowed to adjust the probe’s orientation using a mouse within 4,000 ms. The visuomotor coordination performance was indexed by the orientation difference between the response and the target. The ITI was jittered from 1,000 to 1,300 ms. Participants completed 112 trials in total.

# Learning effect in the WM precision task

Participants performed 20 blocks and 56 trials each block in the retro-cue WM precision task. There might be practice benefits or fatigue deterioration over time. We test these effects by splitting the trials into the first and last halves and by checking whether there were differences in behavioral performance and retro-cue evoked CDA between the two halves. Repeated ANOVAs with Cue (valid, neutral) and Time (first, last) were performed on recall errors and RTs. Participants showed better performance over time (precision: 15.794 ± 3.714 ° vs. 14.788 ± 3.537 °; RTs: 1.297 ± 0.324 s vs. 1.150 ± 0.271 s), supported by the significant main effect of Time on both recall errors (*F*(1, 29) = 10.660, *p* = 0.003, η_p_^2^ = 0.269) and RTs (*F*(1, 29) = 30.590, *p* < 0.001, η_p_^2^ = 0.513). The one-sample *t* tests showed that there were robust CDA components in both the first half (−0.474 ± 0.819 μV, *t*(29) = −3.168, *p* = 0.004, Cohen’s *d* = 0.578) and the last half (−0.728 ± 0.755 μV, *t*(29) = −5.277, *p* < 0.001, Cohen’s *d* = 0.963) of the task. Consistent with the better behavioral performance, the amplitude of CDA in the second half was marginally larger than that in the first half (*t*(29) = 1.910, *p* = 0.066, Cohen’s *d* = 0.349). The statistical analysis of CDA components did not reach a significant level, which might be due to a lower signal-to-noise ratio caused by splitting trials. The amplitude of CDA still highly tracked behavioral recall error in the first hall trials (*r_s_* = −0.402, *p* = 0.028), although it was marginally significant in the second half (*r_s_* = −0.341, *p* = 0.065).

# The exact time points of significant CDA

According to previous studies (Li et al., 2023; Machizawa et al., 2012, 2020; Vogel and Machizawa, 2004;), we measured the CDA component through calculating the contralateral and ipsilateral waveforms relative to the target-located hemifield by averaging across five pairs of electrode sites (P3/4, P5/6, P7/8, PO5/6, and PO7/8). To identify the exact time window of CDA, the contralateral and ipsilateral waveforms were averaged across all participants in the valid cue condition. With analysis package proposed by Luck & Gaspelin (2017), we performed the paired sample t tests between contralateral and ipsilateral waveforms at each time point with false discovery rate (FDR) correction (Benjamini and Hochberg, 1995). The results showed that the time window of significant CDA was from 762 to 1,285 ms after retro-cue onset (Figure S1).


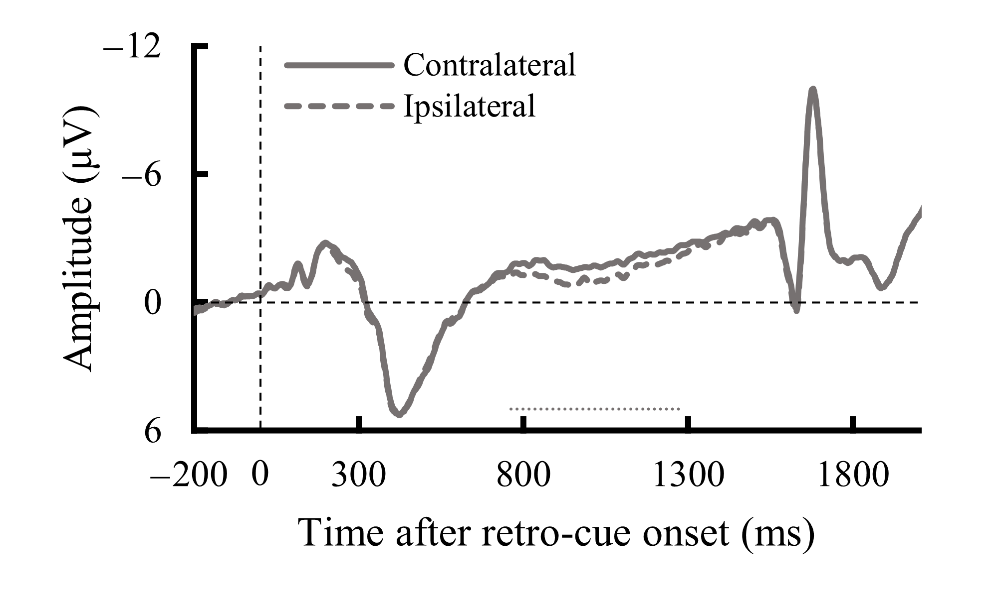


**Figure S1**. The average ERP waveform across all the participants in valid cue condition. The horizontal dotted line indicates 0 μV, and the vertical dotted line indicates the time of retro-cue onset. The contralateral voltage was significantly more negative than the ipsilateral voltage during 762 to 1,285 ms marked by the dotted line.

# Adjusted effect size of the main results

Effect size gives information about the meaningful or practical significance of results and has been suggested to be reported by the *Publication Manual of the* *American Psychological Association,* *6th ed.* (American Psychological Association, 2010). Cohen’s *d* and correlation coefficients have been the most widely reported effect size. Researchers proposed that these effect sizes are sample specific and that they tend to overestimate the population effect size; the adjusted effect size, “corrected” with the sample size, allows for generalization to the population (Ezekiel, 1930; Wang and Thompson, 2007). Our sample is small and we provide the adjusted effect sizes here to indicate the reliability of our results. The adjusted effect sizes were adjusted with the Ezekiel’s formula (1930).

**Table S1.** Unadjusted effect size and adjusted effect size of main results in the manuscript.

| Results | Unadjusted effect size | Adjusted effect size |
| --- | --- | --- |
| Correlation between greater amounts of exercise and better WM precision | −0.437 | −0.403 |
| Correlation between higher frequencies of exercise and better WM precision | −0.483 | −0.454 |
| Correlation between greater total MET and better WM precision | -0.387 | -0.346 |
| Correlation between CDA and behavioral WM precision | −0.380 | −0.337 |
| Group effect on behavioral WM precision | 0.922 | 0.826 |
| Group effect on behavioral CDA | 0.862 | 0.761 |

Note: the exercise represents habitual vigorous-intensity exercise; Unadjusted effect size represents correlation coefficients in the Spearman’s correlation analysis or Cohen’s *d* score in *t* test. Adjusted effect size represents the effect size after adjusting the unadjusted effect size with Ezekiel’s formula (1930).

# References

American Psychological Association (2010). *Publication manual of the American Psychological Association, 6th ed.* Washington, DC: American Psychological Association.

Benjamini, Y., and Hochberg, Y. (1995). Controlling the False Discovery Rate: A Practical and Powerful Approach to Multiple Testing. *J. R. Stat. Soc. Ser. B-Stat. Methodol.* 57, 289–300. doi: [10.1111/j.2517-6161.1995.tb02031.x](https://doi.org/10.1111/j.2517-6161.1995.tb02031.x)

Burnett Heyes, S., Zokaei, N., van der Staaij, I., Bays, P. M., and Husain, M. (2012). Development of visual working memory precision in childhood. *Dev*. *Sci*. 15, 528–539. doi: [10.1111/j.1467-7687.2012.01148.x](https://doi.org/10.1111/j.1467-7687.2012.01148.x)

Cowan, N. (2001). The magical number 4 in short-term memory: A reconsideration of mental storage capacity. *Behav*. *Brain Sci*. 24, 87–114. doi: [10.1017/S0140525X01003922](https://doi.org/10.1017/S0140525X01003922)

Ezekiel, M. (1930). The sampling variability of linear and curvilinear regressions: A first approximation to the reliability of the results secured by the graphic “successive approximation” method. *Ann*. *Math*. *Stat*. 1, 275–315. doi: [10.1214/aoms/1177733062](https://doi.org/10.1214/aoms/1177733062)

Goodale, M. A. (1998). Visuomotor control: Where does vision end and action begin? *Curr*. *Biol*. 8, R489–R491. doi: [10.1016/S0960-9822(98)70314-8](https://doi.org/10.1016/S0960-9822(98)70314-8)

Li, D., Hu, Y., Qi, M., Zhao, C., Jensen, O., Huang, J., & Song, Y. (2023). Prioritizing flexible working memory representations through retrospective attentional strengthening. *NeuroImage*, 269, 119902. [doi: 10.1016/j.neuroimage.2023.119902](https://doi.org/10.1016/j.neuroimage.2023.119902)

Lambourne, K. (2006). The relationship between working memory capacity and physical activity rates in young adults. *J*. *Sport*. *Sci*. *Med*. 5, 149–153.

Luck, S. J., and Gaspelin, N. (2017). How to get statistically significant effects in any ERP experiment (and why you shouldn’t). *Psychophysiology* 54, 146–157. [doi: 10.1111/psyp.12639](https://doi.org/10.1111/psyp.12639)

Machizawa, M. G., Driver, J., and Watanabe, T. (2020). Gray matter volume in different cortical structures dissociably relates to individual differences in capacity and precision of visual working memory. *Cereb*. *Cortex* 30, 4759–4770. doi: [10.1093/cercor/bhaa046](https://doi.org/10.1093/cercor/bhaa046)

Machizawa, M. G., Goh, C. C. W., and Driver, J. (2012). Human visual short-term memory precision can be varied at will when the number of retained items is low. *Psychol*. *Sci*. 23, 554–559. doi: [10.1177/0956797611431988](https://doi.org/10.1177/0956797611431988)

van Ede, F., Chekroud, S. R., Stokes, M. G., and Nobre, A. C. (2019). Concurrent visual and motor selection during visual working memory guided action. *Nat*. *Neurosci*. 22, 477–483. doi: [10.1038/s41593-018-0335-6](https://doi.org/10.1038/s41593-018-0335-6)

Vogel, E. K., and Machizawa, M. G. (2004). Neural activity predicts individual differences in visual working memory capacity. *Nature* 428, 748–751. doi: [10.1038/nature02447](https://doi.org/10.1038/nature02447)

Wang, Z., and Thompson, B. (2007). Is the Pearson r 2 biased, and if so, what is the best correction formula? *J*. *Exp*. *Educ*. 75, 109–125. doi: [10.3200/JEXE.75.2.109-125](https://doi.org/10.3200/JEXE.75.2.109-125)
